# Freeform generative design of complex functional structures

**DOI:** 10.1038/s41598-024-62830-5

**Published:** 2024-05-24

**Authors:** Gerald G. Pereira, David Howard, Paulus Lahur, Michael Breedon, Phil Kilby, Christian H. Hornung

**Affiliations:** 1 CSIRO Data61, Private Bag 10, Clayton South, VIC 3169 Australia; 2 CSIRO IMT, Private Bag 10, Clayton South, VIC 3169 Australia; 3 CSIRO Manufacturing, Private Bag 10, Clayton South, VIC 3169 Australia

**Keywords:** Computer science, Engineering

## Abstract

Generative machine learning is poised to revolutionise a range of domains where rational design has long been the de facto approach: where design is practically a time consuming and frustrating process guided by heuristics and intuition. In this article we focus on the domain of flow chemistry, which is an ideal candidate for generative design approaches. We demonstrate a generative machine learning framework that optimises diverse, bespoke reactor elements for flow chemistry applications, combining evolutionary algorithms and a scalable fluid dynamics solver for in silico performance assessment. Experimental verification confirms the discovery of never-before-seen bespoke mixers whose performance exceeds the state of the art by 45%. These findings highlight the power of autonomous generative design to improve the operational performance of complex functional structures, with potential wide-ranging industrial applications.

## Introduction

Machine intelligence is poised to revolutionize a diverse range of domains. Impact will be keenly felt in the general area of design, and arguably with specific impact industrial design, where generative AI has a critical role to play in creation of a new wave of never-before-seen functional structures that can simultaneously (i) provide a differentiated competitive advantage through superior performance and (ii) contribute to meeting challenging net-zero and carbon reduction targets. Impact will be most prominently felt in domains where the design landscape is challenging and unintuitive, and where manual designs are the current de facto solution.

One such prominent domain is flow chemistry, which over the past decades has been established as a low-barrier-to-entry enabling technology for high-value-add & low-volume chemical manufacturing, which has revolutionised key sectors that traditionally resorted to batch operations. Flow chemistry can add a level of process intensification, commonly associated with cost and energy savings, unavailable to other methods. Flow chemistry offers improvements in yield and a reduction of reaction time^1^, whilst also being more scalable, controllable, greener^2,3^, and generating fewer unwanted byproducts than batch operations^4^. Significant impact has been evidenced in the uptake of flow chemistry across diverse reaction domains, including electrochemistry^5,6^, photochemistry^7^, hydrogenation^8^ and others^9^. These reaction classes make flow chemistry a great test case for a generative AI solution, as they span a wide range of fluidic systems, each of which presents a unique set of requirements for the design of a performant reactor, (typically optimizing of mixing and heat & mass transfer). Combined with additive manufacture^10,11^, this has unlocked huge potential to develop bespoke reactor geometries tuned to the specifics of each reaction type and application.

Given the apparent diversity in chemical reaction domains and demands for flow chemistry, as well as the relatively ‘plug and play’ nature of the performance-governing mixing element itself (herein termed a *static mixer*), it is perhaps surprising that most flow reactions are currently undertaken using one of a highly limited set of commercially available static mixers^12^, e.g., the Kenics^13^, SHM^14^, and SMX^15^ mixers, which have low surface area compared to the CSIRO benchmark^6^. These are all hand-designed, based on a combination of simple, repeated geometric primitives, and largely relying on the know-how of the designer. Moreover, designing improved static mixers is incredibly challenging—the relationship between mixer geometry and emergent flow properties is unintuitive, and it is difficult to assess how modifications to a given design affect performance or other desirable properties. In other words, it is highly unlikely that *any* of the industry adopted standard mixers are optimal for their given application. The mismatch between the diversity of required performance profiles, and small set of hand-tuned mixing options, is a critical ongoing issue in the field resulting in limited mixing performance and a constrained set of attainable reaction types.

To date it has been unclear how to unlock the promise of bespoke mixing. There are numerous reasons for this. Primarily, the design space is unintuitive, multimodal, and difficult to navigate, and there is a lack of design tools to provide a variety of novel and high-performance geometries across a broad design space. Coupled to this, many implementations of fluid dynamics simulation are computationally costly, limiting the number of possible evaluations that can be used to explore the design space. Finally, freeform manufacture of these elements at a feasible resolution has only recently become viable thanks to advances in additive manufacture.

Computational approaches^16^ have shown some improvements over baseline mixers by coupling a gradient-based optimiser to a computational model^17^, focusing on parametric optimization of geometrically simple families of designs^4,15,18^—crossing bars or rotated layers of repeating X-shapes. Limited gradient-based shape optimization^17,19^ is also seen, however many interesting and potentially impactful variables are fixed and not subject to optimization. Additive manufacture, particularly using metals, provides a direct route towards instantiation of customized mixers, however work to date has largely focused on adding features such as tempering channels for heat dissipation, rather than computational design optimisation^20^. Recent research^21^ couples gradient-based mixer optimization via immersed boundary method^22^ with additive manufacture via Electron Beam Melting (EBM). Performance is experimentally verified, and printability is considered, however optimized design differs from the reference geometry only through the size of the constituent bars leading to a simple, constrained design space that is typical of modern approaches. Combined with a predominantly simulation-only verification, performance improvements to date have been relatively modest, and the state of the art in mixer design has not progressed far beyond the aforementioned hand-designed benchmarks for practical applications.

It is fair to say that vast swathes of the possible mixer space remain untouched and cannot be accessed following the above approaches. We are therefore motivated to unlock the potential of mass transfer optimized flow chemistry by *providing a generative design tool for bespoke mixing elements*, addressing a critical unmet need to match increasing reaction performance and efficiency demands and allowing flow chemistry to support a diversity of new, industrially relevant application domains.

## Generative mixer discovery

**Figure 1:**
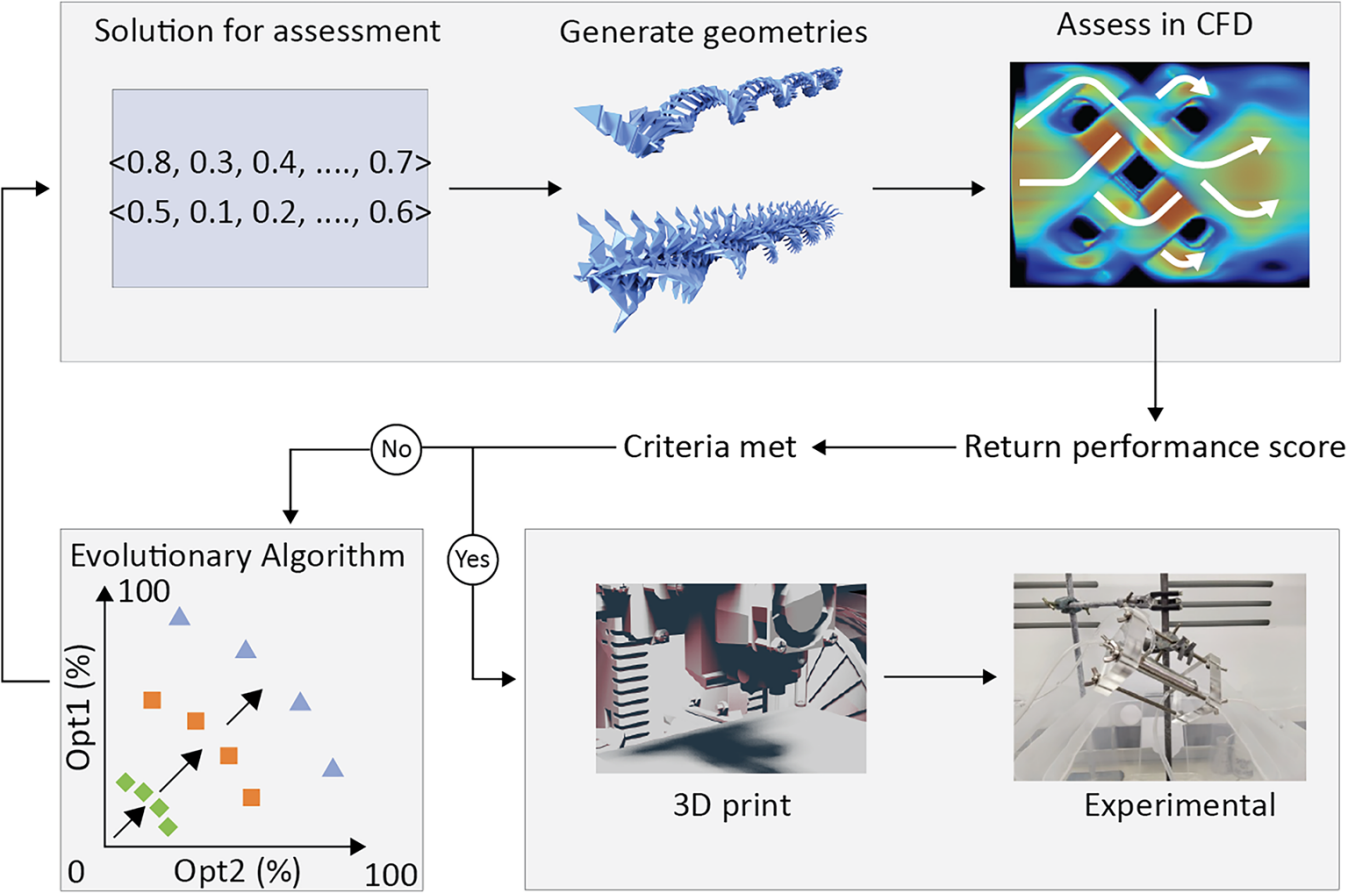
End to end workflow for the design of novel static mixers. Solutions are represented as strings of floating-point numbers, which are transformed into a solid body geometry whose expression depends on the representation used. Geometries are assessed in a Computational Fluid Dynamics solver, and two performance metrics are measured. The main loop feeds these metrics back into an Evolutionary Algorithm which iteratively improves the set of optimal trade-offs between these objectives. If termination criteria are met, the final designs are 3D printed and their performance experimentally confirmed.

We describe a scalable, end-to-end design framework that iteratively explores an expansive, previously untapped design space of bespoke mixing elements. Our workflow is overviewed in Fig. 1 and fully described in the Methods section. Mixers are computationally optimised and can be easily 3D printed and inserted into a given flow chemistry process. Contrasting the state of the art, our approach hinges on an expressive Evolutionary Algorithm (EA)^23^ that produces libraries of designs^24^, and a set of novel representations (mappings between genetic number vector and final geometry) that provides a wide range of attainable static mixer geometries. As black box algorithms, EAs are representation-agnostic, and can be easily tweaked to optimise arbitrary numerical fitness measures with no implicit constraints on the final design morphology. This freedom befits the plug and play nature of flow reactors as well as providing applicability across a range of useful mixing domains. Moreover, it promotes the generation of novel reactor designs^25^. EAs are proven to work in challenging design domains^26^ including morphology generation in soft robots^27,28^, biological ‘xenobots’^29^, and evolving in reality^30^, as well as providing leading designs in engineering domains^31,32^, including structural design^33^ that can be physically instantiated^24^.

Rapid parallel fluid dynamics simulation^34^ provides performance feedback to the evolutionary algorithm and informs future design iterations. Discovered mixers can be easily instantiated in a physics model, or 3D printed and experimentally verified. Experimental verification confirms the discovery of never-before-seen bespoke mixers whose performance significantly exceeds the state of the art, indicating potential broad application of our methodology^35^.

## Results

### Model-based design exploration

We evolve mixers for optimized chemical adsorption of target components from the mobile phase to the mixer surface. We focus on the removal of copper ions from water, which is a vital technology contributing to water security. Note that our experimental design optimizes the general case of adsorption—we do not tune our algorithm to any specific adsorption reaction and as such is theoretically generalizable and transferrable across a range of high-value reactions.

The mixer forms one electrode of the electrochemical cell on which copper ions are adsorbed, and then extracted from solution. It is separate from the outer cylindrical casing which forms the counter electrode. The outer diameter of the inner electrode (mixer) is 6 mm, while the outer cylindrical counter electrode has a diameter of 20 mm and length of 120 mm. These dimensions are common between our model and experimental setups.

We explore the mixer design space by iteratively generating new candidate solutions, which are represented by a vector of real numbers in the range [0–1]. Each time an assessment is required, the mixer’s “genome” (number vector) is transformed into a solid geometry, and then into a voxelised representation suitable for running in our Lattice-Boltzmann Fluid Dynamics solver (Fig. 1).

We maximise reaction efficiency by simultaneously optimising two objectives. The first measures the fraction of virtual tracers that intersect with the mixer surface (Opt1: Transport to Substrate), and the second measures the overall uniform distribution of tracers along the length of the reactor (Opt2: Bulk Mixing), which are both expressed as percentages. Our algorithm generates a Pareto Front (set of optimal trade-offs) between these two competing objectives. This allows researchers to pick solutions from the front that meet their specific requirements.

Once all objective scores for a given iteration (generation) are returned to the evolutionary algorithm, a new set of candidates is generated, and the next generation begins. In the reported experiments, each iteration creates 16 new candidates and is run for 150 generations on a high-performance compute cluster. This number was selected based on pre-experimental trials that assessed average convergence times for the problem, where convergence is defined as a lack of fitness improvement in the pareto front over 10 generations. The mean value was ~ 100 generations; 150 was therefore selected conservatively to account for variance in convergence times due to algorithm stochasticity. These numbers can be tailored to available compute resources. To account for the stochastic evolutionary process, each optimization is repeated five times. Full parameter settings for the evolutionary algorithm are provided in the Methods section.

To illustrate the flexibility of an evolutionary approach, we investigate two representations that map the genome (number vector) to output geometry, allowing us to access different regions of the mixer design space.

### Representations

**Figure 2:**
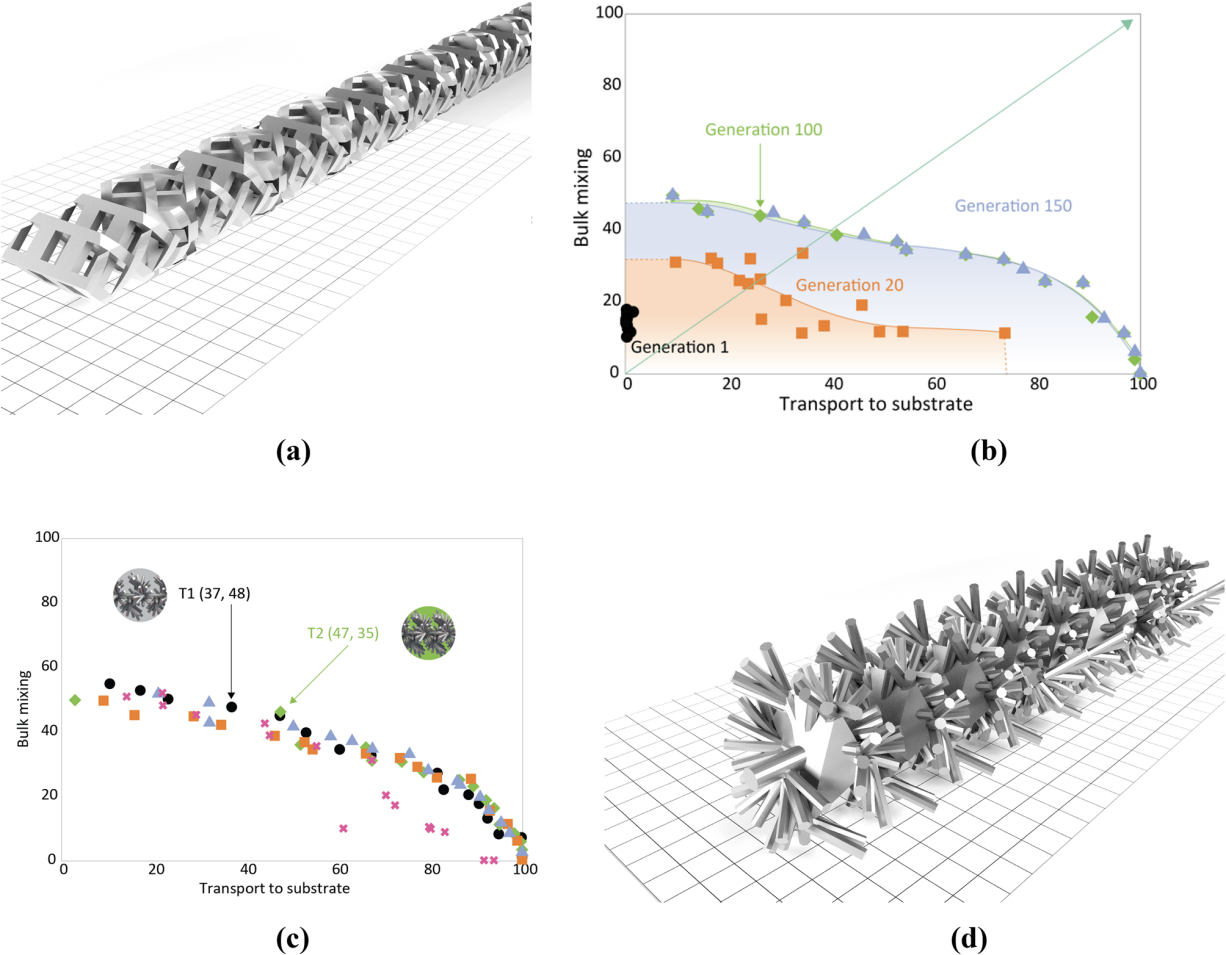
Results of model-based optimization using the Tree (T) representation. (**a**) Illustrating the comparative CSIRO V2 mixer (used for experimental comparison only, included here to highlight differences in geometry and complexity compared to our mixers. (**b**) Progression of the Pareto front in a single experimental repeat over 150 generations, with a theoretical (unattainable) maximum performance at (100, 100). Significant performance improvements are observed over the first 100 generations, with convergence at 100–150 generations. (**c**) Final pareto fronts over the five experimental repeats (one colour/shape per repeat). Highlighting two particularly promising mixers, T1 and T2, with their optimization values shown in parentheses. (**d**) The final geometry for T1.

The tree representation (T) produces bioinspired tree-like structures. Results using the T-representation are shown in Fig. 2. Figure 2a shows the geometry of the CSIRO V2 mixer, which is used as a state-of-the-art baseline that outperforms the other standard mixer types for this class of reaction (*6*). Figure 2b shows a typical progression of the Pareto front from generations 1 to 150, noting the discovery of the optimal front within approximately 100 generations. The initial generation displays negligible Transport to substrate values and low Bulk mixing (< 20). By the twentieth generation (red squares) the pareto front has expanded to a wider range of Transport to substrate (around 75) and Bulk mixing values (up to 35). By generation 100 (green diamonds) this Pareto front has further expanded out for both Transport to substrate (100) and Bulk mixing (up to 50). The final converged Pareto fronts (Fig. 2c) show good agreement, and an illustrative evolved tree-like geometry, T1, is shown in Fig. 2d.

**Figure 3:**
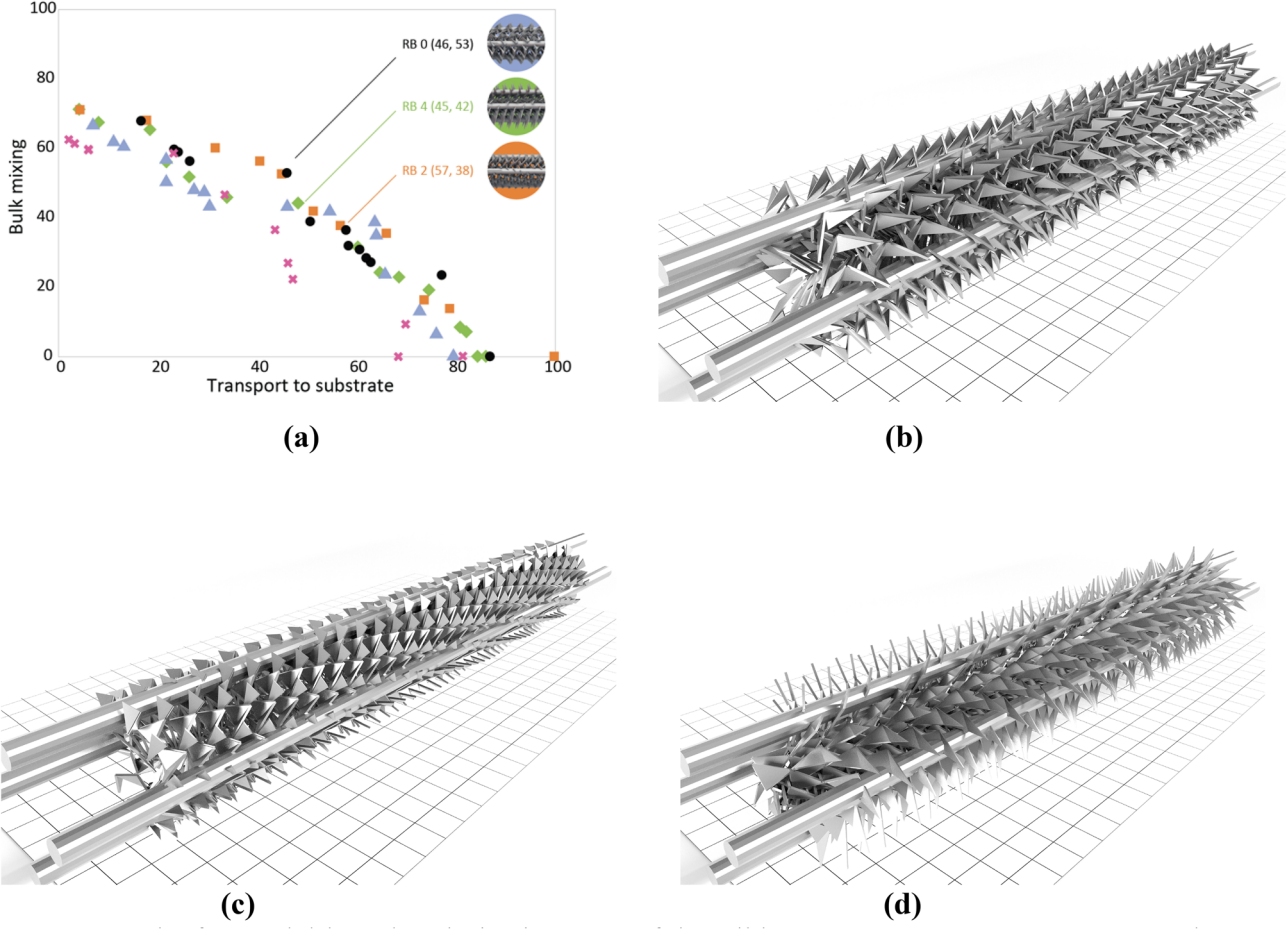
Results for model-based optimisation of the Ribbon geometry (RB). (**a**) Converged Pareto fronts across the five experimental repeats highlighting three discovered high-performance mixers. (**b**–**d**) Geometries of the mixers: RB0, RB2, and RB4 respectively.

Our second representation, the Ribbon (RB), parametrically specifies a series of points in three-dimensional space which are subsequently meshed into a variety of different ribbon-like geometries. Figure 3a shows the final Pareto fronts over the 5 experimental repeats—compared to that of the Tree representation, the Bulk mixing measure generally reaches larger values (up to 70), however the maximum Transport to substrate value approaches 85 (compared to 100 for the T representation): generally, RB geometries promote bulk mixing at the expense of transport to substrate. Figure 3a highlights several high-performing solutions, of which three: RB0, RB2, and RB4, are shown in Fig. 3b–d respectively.

### Experimental verification

**Figure 4:**
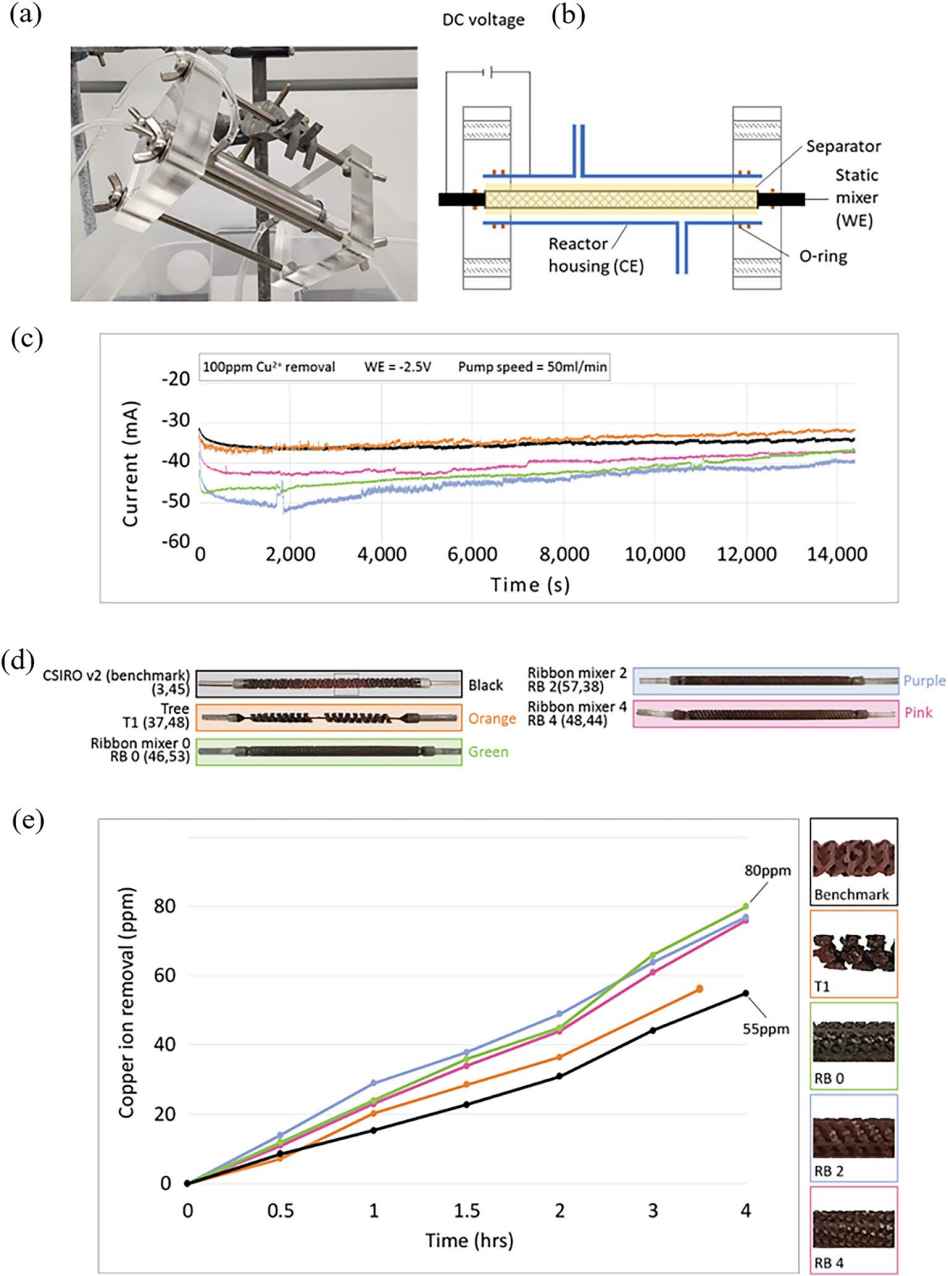
Results of experimental confirmation of mixer performance. (**a**) image of electrochemical flow cell and (**b**) schematic diagram. (**c**) Chronoamperometric response of the experimentally evaluated static mixers in the electrochemical flow reactor, (**d**) photographs of each 3D printed static mixer after extraction from the benchtop experimental system—optimisation values attained via CFD assessment are shown in parenthesis, and (**e**) their corresponding copper ion removal rate as determined by ICP-OES. Background colours in the images correspond to line colours in the plots. All evolved mixers exceed benchmark performance.

We experimentally confirm the performance of a number of promising mixers (those that achieve high values for both optimisation metrics) extracted from the Pareto fronts of both T and RB representations—T1, RB0, RB2, and RB4. The voxelised form of each mixer was 3D printed in stainless steel (316) via Electron Beam Melting on an Arcam A1 3D printer, and run in a benchtop experimental setup. Our mixers are compared to the CSIRO Static-Mixer Electrode, a high-performing benchmark representing state of the art performance for this reaction type, previously described in the literature^6^, and denoted here as CSIRO v2 mixer, with experimental performance in our benchtop experimental setup (see Fig. 4a and b) agreeing with that reported in previous work.

The corresponding chronoamperometric response of each static mixer is presented in Fig. 4c, noting that the RB mixers supported a relatively higher steady state current than the CSIRO v2 mixer over the first 4 h of testing. T1 also outperformed the CSIRO v2 mixer, with a cross-over point occurring at approximately 1800s where the steady state current is lower than the reference. Generally, increased current magnitude corresponds to increased reduction of copper, therefore high performing mixers display higher steady state current. However, because the current reported in the figure is a function of the electrolysis of water as well as the reduction of copper, additional experimentation is required to fully isolate the reduction effect.

The removal of copper ions from solution forms a dark coating on the static mixer (Fig. 4d). The Cu^2+^ removal rate as a function of time (Fig. 4e) is a conclusive measure of electrochemical performance, which was achieved through the removal of an aliquot of reticulated testing solution for further ICP-OES analysis against known analytical standards. The electrochemical flow reactor was configured to promote the electrochemical removal of copper ions.

Over a four-hour period, the CSIRO v2 mixer recovers the least amount of copper from solution of any static mixer evaluated, removing 55 ppm Cu^2+^ from solution. Interestingly the T1 mixer performs marginally better than baseline, while the three ribbon mixers all perform approximately equivalently and significantly better than the baseline, (approx. 80 ppm after four hours). RB mixers were approximately 45% better at removing Cu^2+^ from solution than the CSIRO v2 mixer under identical electrochemical flow conditions.

A complete comparison of the five static mixer designs is shown in Table 1. Substrate transport and bulk mixing values are calculated by the Lattice Boltzmann method, The CSIRO v2 mixer has a low substrate transport, which is reflected in the 55 ppm Cu^2+^ ion removal rate after 4 h of operation. Despite having a 20% lower surface area than the reference, the T1 mixer exceeded baseline performance for substrate transport. Unlike T1, the RB mixers had a substantially higher surface area (~ 70%) than the reference mixer, which was coupled with improved substrate transport characteristics over the reference and T1, which also corresponded with enhanced Cu^2+^ ion removal during after 4 h of operation.

**Table 1:** Showing key static mixer metrics.

Mixer reference	Substrate transport (%)	Bulk mixing (%)	Area (mm^2^)	Surface area change (%)	Copper ion removal at 4 h (ppm)
CSIRO v2 (Reference)	3	45	2126	–	55
Tree (SK1)	37	48	1699	− 20	61
Ribbon (RB0)	46	53	3672	+ 73	80
Ribbon (RB2)	57	38	3715	+ 75	77
Ribbon (RB4)	48	44	3587	+ 69	76

All mixers show improved performance over the benchmark mixer. Tree geometries display reduced surface area (calculated with respect to the reference mixing element). Ribbon geometries display the most improved performance coupled with significantly increased surface area. Given these differences, selection of a mixer representation can be determined depending on the requirements of the target application.

## Discussion

This article describes a generative AI approach to the optimization of complex functional structures (static mixers), with the potential to increase performance and reduce the carbon footprint of a range of different reactions. All four evolved mixers showed better performance than the benchmark CSIRO v2 mixer, which we note was manually designed for the electrochemical reaction we used for our evaluation—this is despite using generic optimisation metrics. The best shapes showed a 45% improvement in copper extracted from solution over a four-hour period, which validates our workflow for this application and as a proof-of-concept indicates this procedure can improve performance of static mixers for a given application. The two representations allow us to explore different regions of the design space of static mixers, with notable differences in terms of surface area and optimisation values. The plug and play nature of our framework makes this simple; the optimisation criteria, evolutionary algorithm, and representation can be freely replaced as required for specific application domains.

As we generate Pareto fronts, which are libraries of varied solutions, there is significant opportunity to use already-discovered mixer designs for specific application domains without running further evolution. In other words, there is a good chance that we have already discovered mixer(s) within the Pareto fronts that display the required balance of *Bulk Mixing* and *Transport to Substate* for a given problem (provided favourable settings for these values are known a priori).

We note some limitations to our approach. First, the reported (simulated and experimental) reactions all occur in the laminar regime. Turbulent flow may be less well-behaved and require further workflow development to create solutions for such regimes, however we note that operating in a turbulent flow regime is not necessarily a precursor to enhanced reaction performance^36^. Secondly, the 3D printing process also creates fabrication noise, primarily observed as surface roughness, which is a function of the precision of additive manufacturing process employed in the instantiation process. Further investigations are required to assess the effects on performance, as roughness may be beneficial to mixing and it may be preferrable to keep or even induce texture on the mixer’s surface rather than eliminate it through more accurate fabrication processes.

Finally, we highlight the ability to integrate our bespoke reactor generation algorithm with AI and robot-controlled flow chemistry setups^37,38^, providing an adaptive method of customising the physical apparatus of the experiment, as well as the potential to combine with other frontier technologies to continue to push the boundaries of high-performance chemistry^39^.

## Methods

Our generative design^40^ workflow can be described in terms of its constituent components: numerically represented mixers are expressed as 3D models, evolved to maximise multiple objective functions, and assessed via rapid fluid dynamics. The final best designs are 3D printed and their performance is verified.

### Representation

We investigate two different representations (mappings between number vector and STL geometry), highlighting the types of design space that can be successfully traversed using this approach.

Each individual is represented by a finite vector of real numbers in the range 0–1 and is interpreted to output a finite set of simple polyhedrons, where each polyhedron consists of a finite set of simple polygons. Simple polygons and polyhedrons do not self-intersect. However, a polyhedron may intersect or even fully contain another polyhedron. Practically, we deal with issues around invalid (self-intersecting) STLs by voxelising (the voxelised input is used for both computational modelling and 3D printing; see later).

Our application calls for exploration of a large design landscape with the potential for candidates that human designers typically do not consider. Here, we study two families of geometries inspired by well-known biological or geometric phenomena. Aside from generating novel flow reactors, the use of two differentiated representations highlights the potential for ‘plugging in’ other representations to access different regions of the design space. For this work, our representations are (i) a tree-like (T) geometry, and (ii) a ribbon-like (RB) geometry. Note that these geometries are significantly more complex than any current hand-designed mixing elements.

### Tree-like geometry

The Tree-like (T) geometry is a bioinspired representation^41^ based on Lindenmayer systems^42^. T geometries are constructed from a base ‘trunk’, from which branches of increasingly smaller size are recursively created. Because branches at all levels are constructed through the same algorithmic process, a small set of design parameters can map into a family of complex shapes. The elemental shape of the tree is a simple cylinder. Cylinders may overlap each other in the STL form, as the voxel grid generator is guaranteed to produce valid geometries for both modelling and printing. T geometry genomes consist of ten real numbers that fall into the range of [0.0, 1.0]. These generic values are then linearly mapped to a set of parameters in the range [*x*_min_, *x*_max_], where x is a given allele and each allele is either a real number, integer, character or Boolean. For T geometries, Table 2 shows the role of each parameter.

**Table 2:** Parameter definitions for the T representation.

Param. No	Parameter role	Min value	Max value	Note
1	Trunk length			Unused in this rep
2	Trunk diameter			Unused in this rep
3	Branch count	5	Int (trunk length)	The number of branches growing on the trunk
4	Branch length	1.0	3.0	
5	Branch diameter	0.3	Trunk diameter	
6	Branch angle	0.0	Pi	0: in the same direction as trunk, Pi/2: perpendicular, Pi: opposite
7	Max level of branching	1	3	1/2/3 levels of recursion
8	Twig count	1	2 * int (round (branch length))	
9	Diameter branching factor	0.6	1.0	
10	Length branching factor	0.5	1.0	
11	Count branching factor	0.5	2.0	
12	Angle branching factor	0.5	1.0	

“Branching factor” is used to calculate the properties of a branch with respect to its parent branch. For example, a branch with diameter of 0.4 and “diameter branching factor” of 0.7 will create twigs of 0.4 * 0.7 = 0.28 diameter, which in turn will have smaller twigs of 0.196. The remaining parameters to generate the tree can be computed from these parameters listed above. One of them is the linear spacing between consecutive branches on the trunk, which is set to be uniform along the trunk length. This also applies to smaller branches. Similarly, angular spacings between consecutive branches on the trunk are set such that all branches cover a full circle uniformly.

Initial experimentation revealed that incorporating additional shapes improved mixing performance, by increasing potential surface area. This is achieved by connecting the base of twigs (the smallest branch in this set up) of the same parent branch into a convex hull. Note that this shape is formed only when there are at minimum four twigs to connect, which results in a tetrahedron.

### Ribbon-like geometry

Our second family of geometries are based on ribbons (RB). RB geometries produce more variety and are correspondingly less predictable in the outcome. To achieve this, higher degrees of design freedom are required, which requires a higher-dimensional parameterization than that of the T geometries. Again, each parameter is a real number that falls into the range of [0.0, 1.0]. These generic parameters are then linearly mapped to parameters *x* with certain type and range to suit their specific roles. RB geometries have 30 input parameters, shown in Table 3.

**Table 3:** Parameter definitions for the RB representation.

Param. no	Parameter role	Min value	Max value	Note
1–27	Vectors	0.0	1.0	9 vectors in 3D to define unit ribbon
28	Spacing between unit ribbons	0.1	2.0	Spacing along mixer length
29	Twist angle between unit ribbons	0.0	2.0*PI	To achieve helical configuration
30	Reserved			Currently unused

Starting from the point of origin, a unit ribbon is constructed using by stringing the 9 vectors together, resulting in 10 consecutive points where **P**_1_ = (0, 0, 0) and **P**_i+1_ = **P**_i_ + **V**_i_. These points are mapped into a triangulated mesh, where triangle T_i_ is defined by the three points **P**_i,_ **P**_i+1,_ **P**_i+2_. The result is a set of interconnected triangles that form a ribbon. As the last step in the construction of a unit ribbon, these triangles are converted into prisms by adding a small, predetermined thickness. This unit ribbon is then replicated along the longitudinal axis of the mixer, with parameterised interval and twisting angle around the axis.

Surface geometries are written out in STL (Stereolithography) format, which is the most common format in 3D printing. Since fluid dynamics assessment in the following step requires a 3D Cartesian grid consisting of a set of voxels (cubes), the current step, which is called voxel grid generation, uses the surface geometry to determine which voxels are fluid, and which are solid. This is done by first flagging the voxels that intersect the surface geometry as “solid”, resulting in multiple contiguous groups of unflagged voxels. Then, the group of voxels that contains certain predefined points in space is set as “fluid”. All the remaining voxels are subsequently flagged as “solid”. Finally, voxels that fall outside of a predefined computational domain boundary are flagged as “solid”. In this case, the mixer is housed inside a cylinder with the dimensions: diameter 6.24 mm, length 48 mm, and total volume is approximately 1468 mm3.

**Figure 5:**
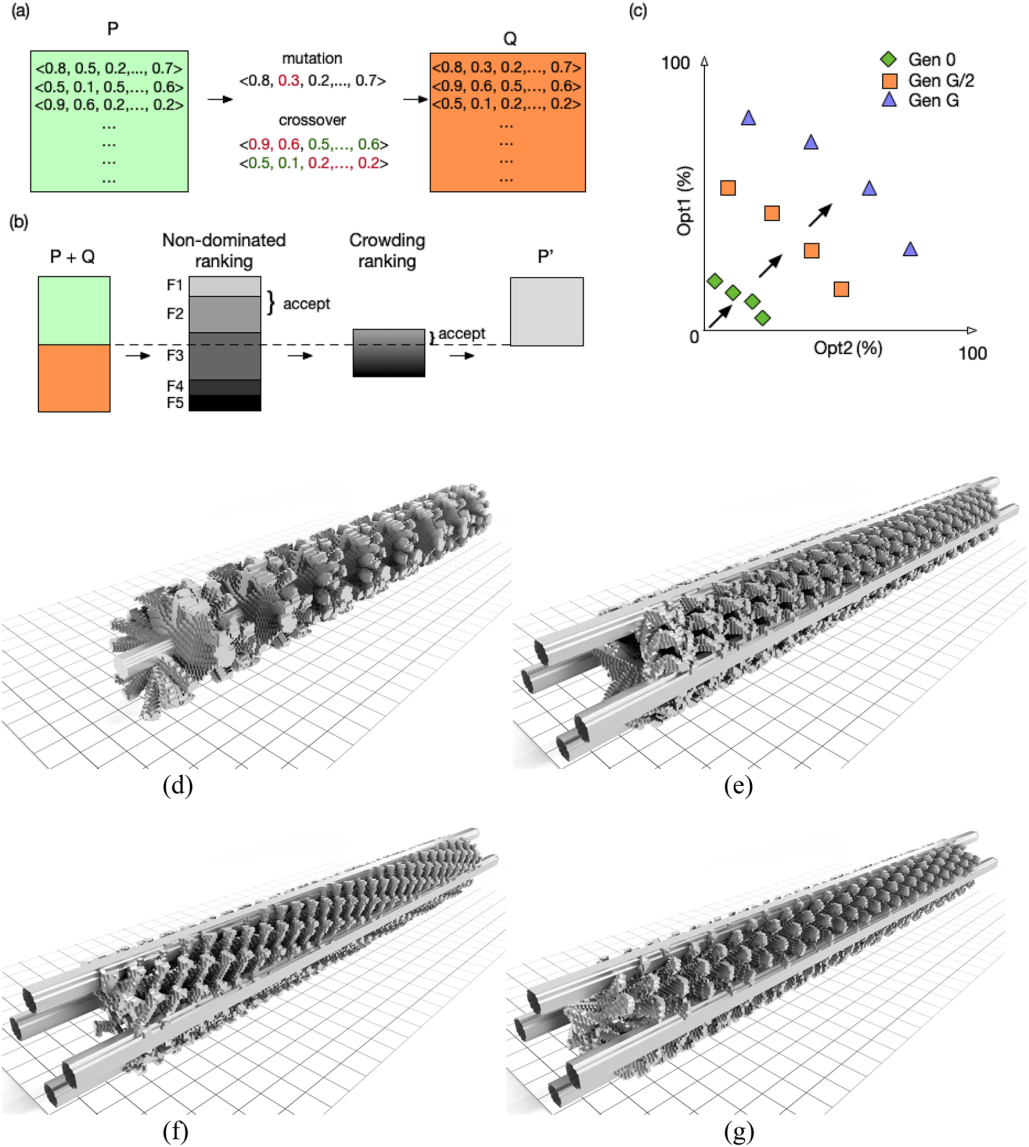
Describing the evolutionary algorithm NSGA2. (**a**) A set of new candidates Q is generated from current population P using mutation and crossover operators, in this case showing point mutation and one-point crossover. (**b**) P and Q are combined and sorted into fronts F based on dominance. Fronts are accepted into P’ in order; the front that causes the size of P’ to exceed the size of P is ranked based on crowding distance (Manhattan distance between points in fitness space), and individuals accepted until sizes of P and P’ are equal. (**c**) Through G generations, the set of Pareto optimal solutions incrementally improves so the final Pareto front contains a set of optimal trade-offs between the two objective functions Opt1 and Opt2. We additionally show the voxelised forms of four promising geometries created through this approach. This is the representation used in both the CFD and for 3D printing. Mixers are (**d**) T1, (**e**) RB0, (**f**) RB2, and (**g**) RB4.

In addition to 3D volumetric grid, the process above also produces the interface between fluid and solid regions, which is written out in STL format. While one might consider this a rough approximation to reality, note that these mixers will ultimately be 3D printed and the voxel resolution of the 3D printer is set equal to the voxel size for the grid discretization. Voxel size is set to 120 microns, which is accurately reproducible with our 3D printer with minimal warping but may be adapted depending on specific fabrication constraints. The voxelised T1, RB0, RB2, and RB4 mixers used for experimental assessment are shown in Fig. 5.

### Fluid dynamics assessment

Once the voxelised form of the mixer has been determined it can be used to directly solve important fluid flow properties using Computational Fluid Dynamics^43,44^, a widely applicable technique for various fluid-based^45,46^ modelling problems. We use the Lattice Boltzmann (LB) method^47^, which is rapid, easily parallelized and voxel-based. LB is particularly suited for our generative design framework, as (i) the grid on which the LB method is solved directly maps to the voxelised geometry for the 3D-Printer, (ii) The CAD geometries supplied for the various mixers do not require meshing which can be problematic for some other (more classical) CFD methods, especially considering the types of meshes we generate (see Figs. 2 and 3). Additionally, (iii) complex and irregular boundaries can be easily handled^34^. An inlet and outlet are included in the model which allows us to numerically calculate the pressure drops and velocity fields within the mixer. For the present study we only consider the flow of a single fluid and analysis of experimental flow conditions reveal Reynolds numbers well within the laminar flow regime.

The voxelised mixer is imported into the CFD and fluid flow simulated until the velocity field reaches a steady-state condition. To reach this condition, average flow velocity over a sequence of 3 consecutive cycles, where each cycle is 1000 simulation time-steps, must be less than a dimensionless threshold value of 1.0E-3. At this point the simulation is terminated. Exact time to achieve the steady-state condition depends on mixer geometry, ranging between 600 s and 1200 s.

Once this flow field is established, a multitude (+ /− 100,000) of massless virtual tracer particles are released from the inlet and their paths traced through the mixer. Both of our objective values are derived from the performance of these tracers, which may flow in a number of ways that have a significant effect on mixer performance, e.g.; (i) towards a solid substrate and end up adjacent to this substrate, in which case the particle would enter the no-slip (velocity boundary condition) region and display extremely long residence time, (ii) in an eddy/vortex so that its translational velocity is negligible (again displaying a long residence time), or (iii) to the outlet.

Tracers are advanced using the local steady-state velocity field, ***v***, generated by the CFD^48^. Tracer position is updated via Eq. (1), which is integrated using a fourth order Runge–Kutta scheme with the present position of the tracer denoted as ***r***(t).

1 $$\frac{{d{\varvec{r}}\left( t \right)}}{dt} = {\varvec{v}}\left( {\varvec{r}} \right)$$

The tracers, which begin at the inlet, will move a fraction of a voxel size every time-step. However, the LB method calculates the velocity at (discrete, integer) lattice points. Trilinear interpolation is therefore used to obtain the velocity field at the precise position where a tracer is located (which is typically not at a discrete lattice point), using velocities from the neighbouring 8 lattice nodes. As the particles only move according to the velocity field only fluid advection is considered. Since “chaotic advection”^49^ is the goal of design of these mixers, neglecting fluid diffusion is appropriate.

Tracer maps are constructed from these trajectories to statistically determine the mixing and adsorption measures outlined below. As a statistically significant number of tracers are required to create accurate fitness measures, the tracer mapping part of the CFD is computationally expensive (approximately 2.5 h wall time per assessment, for a total of around 3 h wall time including attaining a steady state simulation and subsequently performing tracer-based assessment). Shortcutting the tracer-based assessment would remove a significant amount of veracity from the performance assessment, therefore we instead elect to spread the computational effort across an HPC system, maintaining accuracy and providing a degree of scalability to our approach.

### Performance assessment

We select two fitness measures that are generic (can be applied to a range of static mixing problems) and informative (provide useful, accurate measures that translate well to reality). Given that orientations of the substrate (parallel or perpendicular to flow direction) have differing effects on adsorption, particularly in a continuous flow regime, we eschew simple well-used metrics based on surface area, instead calculating Substrate Transport as the fraction of tracer trajectories which intersected with the mixer surface/substrate. However, non-uniform build-up of tracers on the substrate is undesirable as it does not utilize the entire substrate area and can induce blockages in the flow. Consequently, our second measure, Bulk mixing, captures the overall mixing of tracers (or tracer map) throughout the entire length of the mixer.

Maximizing fluid transport to the mixer’s surface (substrate) is a commonly desired mixer properly and is our first objective function. We identify all tracers that finish the simulation close to the mixer surface. These tracers are easily differentiated from other tracers in that they have extremely long residence times and have a final position close to a solid voxel. Long residence times occur when the tracers enter a no-slip region (velocity boundary condition) and so have low velocities.

Substrate Transport, *S*_*T*_, is defined as the number of tracers which end up adjacent to the mixer substrate as a ratio of the total number of tracers.

2 $$S_{T} = \left( {{\text{tracer}}\;{\text{adjacent}}\;{\text{to}}\;{\text{substrate}}} \right)/\left( {{\text{total}}\;{\text{tracers}}} \right)$$

We multiply by 100 to give a percentage, where *S*_*T*_ = 100 represents a theoretical optimal substrate transport.

For our second optimization metric, we use a tracer map to obtain a quantitative measure of mixing, known as the coefficient of variation or *Cov*^50^. A cross-section of the CFD simulation is divided into cells. For simplicity square sections with a cell size of 4 voxels are used (determined through initial experimentation). Resultingly, the 6.24 mm mixer diameter was discretized into 51 voxels (120 microns per voxel edge). We then calculate the fraction of tracers in a particular cell (*c*_*i*_) and the mean number of tracers in a cell (*c*_*av*_), as well as the flux of material that passes through that cell^13^ such that cells which have little material passing through them are given lower weighting compared to those with high flux.

*Cov* is calculated as in Eq. (3), where *f*_*i*_ is the flux through cell *i*, total flux through the cross-section is defined as:

3 $$F = \mathop \sum \limits_{i = 1}^{N} f_{i}$$

where *N* is the total number of cells. If all cells contain the same fraction of tracers (i.e., *c*_*av*_), *Cov* = *0*. This corresponds to a homogenous mixture or maximum mixing. Similarly, maximum segregation (i.e., either cells devoid of tracers or cells with maximum tracers) gives *Cov* = 1.

A final note about this mixing measure is that it represents the mixing of bulk fluids. A particular example of this is if two separate fluids entered the mixer (equal amounts in each half cross-section) the mix measure gives a prediction of how well mixed the two fluids are at a particular cross-section down the channel. We express this as a percentage, calculated as (1 − *Cov*)*100.

### Evolutionary algorithm

The Evolutionary Algorithm (EA) is the final part of our model-based optimization loop, which uses the fitness measures calculated from the CFD to iteratively optimize a population of candidate mixers. EAs have a track record in structural optimisation^33,51^. EAs are used as they are flexible, representation-agnostic^52^, and well-suited to operating in highly multimodal search spaces. Furthermore, certain formulations of EAs naturally produce libraries of final solutions, which are a key feature of our approach. Our EA is based on the popular Non-dominated Sorting Genetic Algorithm, NSGA2, which has shown promise in industrial product development^53,54^ and illustrated in Fig. 5a–c.

An initial population of solutions P is randomly generated. That is, each variable is set random-uniformly in the range [0–1], then later expressed as a geometry by mapping to the minimum and maximum values for the specific variable as delineated in Table 2 (for T geometries) and Table 3 (for RB geometries). Each solution is tested in CFD and receives scores for both objective functions.

Next, a set of new candidates Q is generated by copying P. For each candidate in Q, a random unique individual (also in Q) is selected, and one point crossover is performed. A random index between 1 and the size of the candidate genome-1 is selected and the contents of the two candidate vectors are swapped after this point. Each allele in the genome experiences a random perturbation sampled from a normal distribution with a mean of 0 and covariance of 0.1, clipped to the range [0–1].

P and Q are combined and sorted into fronts F based on dominance^53^. Fronts are accepted into P’ in order. If adding a front causes the size of P’ to exceed the size of P, the front is ranked based on crowding distance (Manhattan distance between points in fitness space) and individuals are sequentially accepted into P’, until P and P’ are equally sized. This gives the next population P’. The iterative process of applying evolutionary operators to create Q, assigning objective scores, and finally generating the next population P’ continues through 150 generations. The set of Pareto optimal solutions incrementally improves so the final Pareto front contains a set of optimal trade-offs between the two objective functions. Voxelised examples of high-performing mixers discovered in silico and later experimentally verified are shown in Fig. 5d–g.

## Data Availability

All data that underlies this study is publicly available through the CSIRO Data Access Portal (DAP) with 10.25919/dz2k-mp94, (see 10.25919/dz2k-mp94). The data includes results from all computer workflow experiments we conducted, experimental results, photographic images plus Scanning Electron Microscope images of our Additively Manufactured electrodes. In addition, a directory containing Pseudo-Code of our method is available.
