# Author Correction: Freeform generative design of complex functional structures

**DOI:** 10.1038/s41598-024-66713-7

**Published:** 2024-07-10

**Authors:** Gerald G. Pereira, David Howard, Paulus Lahur, Michael Breedon, Phil Kilby, Christian H. Hornung

**Affiliations:** 1 CSIRO Data61, Private Bag 10, Clayton South, VIC 3169 Australia; 2 CSIRO IMT, Private Bag 10, Clayton South, VIC 3169 Australia; 3 CSIRO Manufacturing, Private Bag 10, Clayton South, VIC 3169 Australia

Correction to: *Scientific Reports* 10.1038/s41598-024-62830-5, published online 24 May 2024

The original version of this Article contained errors in the additive manufacturing method ‘Electron Beam Melting (EBM)’.

As a result, in the Introduction.

“Recent research^21^ couples gradient-based mixer optimization via immersed boundary method^22^ with additive manufacture via Selective Laser Melting (SLM).”

now reads,

“Recent research^21^ couples gradient-based mixer optimization via immersed boundary method^22^ with additive manufacture via Electron Beam Melting (EBM).”

And in the Results section, under the subheading ‘Experimental verification’

“The voxelised form of each mixer was 3D printed in stainless steel (316) via Selective Laser Sintering on an Arcam A1 3D printer, and run in a benchtop experimental setup.”

now reads,

“The voxelised form of each mixer was 3D printed in stainless steel (316) via Electron Beam Melting on an Arcam A1 3D printer and run in a benchtop experimental setup.”

The original Article has been corrected.

